# Demographic Mix of Care Homes and Personalised Use of SGLT-2 Inhibitors and GLP-1RAs in Residents with Type 2 Diabetes Mellitus

**DOI:** 10.3390/jpm16020062

**Published:** 2026-01-28

**Authors:** Alan J. Sinclair, Fiza Waseem, Ahmed H. Abdelhafiz

**Affiliations:** 1 Foundation for Diabetes Research in Older People (fDROP), King’s College, Droitwich Spa, London WR9 0QH, UK; alan.sinclair@kcl.ac.uk; 2 Department of Geriatric Medicine, Rotherham General Hospital, Moorgate Road, Rotherham S60 2UD, UK; fiza.waseem@nhs.net

**Keywords:** older people, frailty, SGLT-2 inhibitors, GLP-1RA, personalised, management, therapy, care homes, diabetes mellitus

## Abstract

Diabetes prevalence in older people residing in care homes is rising. This cohort of patients is characterised by multiple morbidities, polypharmacy, and frailty. As a result, they are exposed to an increasing burden of hypoglycaemia, which leads to unnecessary hospital visits and negative consequences. In addition, due to their high baseline morbidities, the risk of cardiovascular events increases. The newly introduced therapy of SGLT-2 inhibitors and GLP-1RA has a very low risk of hypoglycaemia and a significant cardiovascular protective effect. This makes it an appealing choice to be used in older people with complex morbidities, such as care home residents. So far, the current use of these agents is suboptimal in these settings because clinicians are cautious of side effects and tolerability, and also, clinical studies have not included this population. Furthermore, the guidelines in this area lack a personalised approach and are too general, with no clear specific description of which patients are suitable for such therapy. The currently available little evidence is indirect, which confirms the superior benefits of such therapy in frail compared with robust subjects, especially in those who are overweight or obese. The demographic mix of care homes is largely heterogeneous in terms of variations in body composition. In addition to malnourished, frail phenotype subjects, the prevalence of individuals with obesity living in these settings is increasing. Therefore, there is scope for increased use of these new agents in residents who have at least a normal or higher body weight. Because of the high baseline cardiovascular risk, these patients will benefit most from such therapy. Otherwise, these agents are better when less used for frail patients who are anorexic and malnourished because of the risk of inducing further weight loss, volume loss, low blood pressure, falls, and fractures.

## 1. Introduction

Worldwide, the prevalence of diabetes is expected to rise from 8.4% in 2017 to 10% in 2045 [1]. The rise is specific to older people. For instance, approximately 50% of people with diabetes are over 65 years old, and the peak prevalence (24%) occurs in people 75–79 years of age [1]. With advancing age and duration of diabetes, older people will develop multimorbidities and frailty as a result of the accumulation of conditions and complications associated with diabetes [2]. Multimorbidity and frailty have a prevalence of 80% and 48%, respectively, in older people with diabetes, which increases proportionally with age and duration of diabetes [3,4]. As a result, the phenotype of diabetes in old age is a predominance of multimorbidity and frailty leading to disability and increased risk of institutionalisation. Therefore, an advanced-age population will lead to an increase in the proportion of older people with diabetes living in care homes. The guidelines on hypoglycaemic therapy for residents in care homes are general and non-specific, especially for the use of sodium glucose cotransporter-2 (SGLT-2) inhibitors and glucagon like peptide-1 receptor agonists (GLP-1RAs) [5,6]. These therapies significantly reduce weight, and the guidance is to avoid their use in frail older people at risk of weight loss or intolerance to these agents. However, institutionalised older people with diabetes are heterogeneous, and the use of these agents needs to be personalised. So far, there are no randomised clinical trials of these therapies in care home residents. This is unlikely to happen in the near future due to difficulties in the recruitment of such a vulnerable population. This manuscript reviews the demographic mix and variation in body composition of care home residents, explores the available evidence on the efficacy and tolerability of these agents in this population, and provides a personalised approach for clinicians on which patient groups these agents are suitable for.

## 2. Demographic Mix

Populations in care homes are heterogeneous with a complex demographic mix. There is a high prevalence of frailty and comorbidities, especially among residents with diabetes mellitus. The hypoglycaemia risk is particularly prevalent in these settings.

### 2.1. Diabetes Burden

In care homes, diabetes prevalence is on the rise. In the US, 24.6% of care home residents had diabetes in the year 2004 [7]. Prevalence increased from 1995 to 2004, from 16.9% and 16.1% to 26.4% and 22.2% in males and females, respectively. For very old residents (≥85 years), there was an increase in prevalence from 13.1% to 19.5% in males and from 11.3% to 16.1% in females [8]. A further increase was noted, reaching around 32.8% of residents [9]. A later study showed that among 1409 long-term care (LTC) US residents, diabetes prevalence was 34.2% [10]. A more recent, large cross-sectional study of almost all US care homes reported a similar prevalence of 141,636 residents with diabetes mellitus (33.3% of the total) [11]. In Ontario, Canada, diabetes was prevalent in 35.5% and 36.7% of care home residents in 2017 and 2022, respectively. The prevalence varied geographically and was higher in some areas, reaching from 38.5% to 42.1% [12]. In Europe, a study, which included 4037 residents living in 59 care homes from 8 European countries, reported a prevalence of 21.8% [13]. In a UK study, diabetes prevalence was 19.9% [14]. In a study investigating diabetes prevalence in French Caribbean care homes, the prevalence was 28.3% [15]. The real diabetes prevalence in care homes might be higher due to a lack of regular screening for diabetes development in these settings [16].

### 2.2. Comorbidity Burden

Because of the associated morbidities and complications of diabetes, residents with diabetes living in care homes tend to have a high comorbidity burden. The European Services and Health for Elderly in Long-TERm care (SHELTER) prospective study reported that residents with diabetes have high prevalence of congestive heart failure, ischemic heart disease, stroke, hypertension, and worse self-perceived health compared with those without diabetes [13]. The French care homes study found that residents with, compared to those without, diabetes had a higher prevalence of heart failure (17.0% vs. 7.6%), hypertension (85.1% vs. 59.2%), stroke (24.5% vs. 13.1%), hypercholesterolemia (26.6% vs. 11.8%), kidney disease (22.3% vs. 11.3%), and lower limb arteriopathy (16.0% vs. 8.4%) [15]. Significant levels of disability were found in a retrospective medical records analysis of 75 subjects with diabetes living in UK care homes. For instance, in addition to diabetes, each person had an average of four co-morbidities (range 1–8), and mortality was 34% after one year [17,18]. Older people with diabetes in US care homes have more comorbidities such as kidney failure, cardiovascular disease, pressure sores, visual problems, and limb amputations compared to residents without diabetes [19]. US care home residents with diabetes have more prevalent complications (54% vs. 45%), infections (26% vs. 21%), and emergency department and hospital visits (37% vs. 30%) [10]. Another US study reported that 24% of residents with diabetes were dependent in activities of daily living (ADL) and 58% had dementia [11].

### 2.3. Hypoglycaemia Burden

The major complication of glucose-lowering therapy is hypoglycaemia. In care homes, hypoglycaemia prevalence is estimated at around 28% to 40%, but it was reported to be as high as 41.9% in one study (median episode of 2, range of 1–10 per patient per year) [10,20,21]. Nonetheless, it is possible that hypoglycaemia incidence is underreported. This may be due to the less specific manifestations of hypoglycaemia, such as visual impairment and dizziness, leading to a misdiagnosis [22]. Hypoglycaemia may also mimic dementia, a condition common in care homes, presenting with confusion, agitation, or behavioural changes. Hypoglycaemia increases the risk of hospitalisation, complications, length of hospital stay, and overall costs [23]. Hypoglycaemia risk in care home residents is high due to a high number of morbidities, frailty, dementia, and polypharmacy and the use of therapies with high hypoglycaemic potential, especially insulin [24]. Other risk factors for hypoglycaemia in care homes include drug errors, variable eating patterns and functional dependency [25]. A US care home study found that 43% of people with diabetes had hypoglycaemia, more emergency department or hospital admissions (44% vs. 31%) and mortality (20% vs. 10%) compared with those without hypoglycaemia [10]. Hypoglycaemia is associated with further complications such as falls, fractures and cardiac arrhythmias [26]. Risk factors of hypoglycaemia in care homes are summarised in Box 1.

Box 1 **Hypoglycaemia risk factors in care homes.**

- Old age
- Atypical presentation
- Misdiagnosed as dementia or age-related
- Multiple morbidities
- Organ dysfunction
- Drug errors
- Hypoglycaemia potentiating agents
- Polypharmacy
- Tight glycaemic control
- Malnutrition
- Erratic eating pattern
- Cognitive and physical dysfunction

### 2.4. Frailty Burden

Frailty and pre-frailty prevalence in care homes is estimated at 52.3% and 40.2%, respectively [27]. The frequency of mildly frail residents in US care homes is 7.6%, moderately frail 44.5%, and severely frail 47.9% [28]. In a French study, 54.2% of residents were most frail, and 34.6% were frail [29]. In a Chinese study including 1004 nursing homes, the frailty prevalence was 55.6% and pre-frailty 38.5% [30]. The prevalence of frailty may vary by the frailty assessment tool used. For example, frailty prevalence was 71.8%, 42.7%, and 36.4% according to the Rockwood clinical frailty scale, frailty in nursing home scale, and Fried index, respectively [31]. Using the FRAIL-NH scale in German care home residents, 71.1% were most frail, 26.4% frail, and 2.5% non-frail. The clinical frailty scale (CFS) showed that 6.9% of people were non-frail, 26.8% were mildly to moderately frail, and 66.3% were severely frail. Both scales show good agreement and correlated significantly (r = 0.78; R^2^ = 60%) [32]. Sarcopenia, a condition closely linked to frailty, is also prevalent in care homes. In a systematic review, the pooled sarcopenia prevalence, as defined by the European Working Group on Sarcopenia in Older People (EWGSOP) criteria and skeletal muscle index (SMI)-defined sarcopenia, was 41% and 59%, respectively [33]. Another systematic review found that sarcopenia prevalence is highest in care homes compared with the community and hospitals [34,35].

## 3. Body Composition

The body composition of people in care homes is heterogeneous and spans from individuals with obesity on one side to underweight individuals on the other side. In a Canadian study, a large number of people with diabetes were either overweight (30.3% vs. 28.2%) or obese (27.1% vs. 18.6%) compared with those without diabetes [12]. Studies from Europe reported that residents with obesity were more likely to have diabetes mellitus [15,36,37]. The Slovenian cross-sectional multi-centre NutriCare study found that the prevalence of obesity was 38.3%, sarcopenia was 27.6%, and sarcopenic obesity was 24.5% [38]. A US care home study showed that 41% of residents with diabetes had a BMI ≥ 30 kg/m^2^ [11]. The frequency of obesity in US care homes has been steadily increasing, rising from 22.4% in 2005 to 28.0% in 2015. Overweight, normal weight, and underweight prevalence fell from 28.9% to 27.8%, 40.3% to 37.1%, and 8.5% to 7.2%, respectively [39]. Underweight, which is likely related to anorexia, is also prevalent in care homes. The decrease in appetite that is caused by ageing, rather than an illness, is known as anorexia of ageing, which affects up to 30% of older people in the community and 85% in care homes [40]. It could be related to physiological alterations of energy balance, psychological factors, social isolation, changes in gut hormones, and a reduced sense of taste, which may reduce the desire to eat [41,42,43,44,45]. Anorexia leads to subsequent loss of weight, physical activity, and muscle mass and muscle strength [46]. In a German care home study, the prevalence of underweight was 13.7%, and anorexia was the most important contributing factor [47]. In an Italian study, 15.4% of residents were malnourished [48]. It has been reported that in care home residents, daily intake of protein was 0.80 g/kg body weight, daily intake of energy was 20.7 kcal/kg body weight, and the oldest (>85 y) residents were the most affected [49]. It was suggested that malnutrition and loss of weight prevalent in care homes are linked to the majority of chronic morbidities and health-related issues, with the exception of cardiometabolic diseases [50].

It appears that older people living in care homes are a heterogeneous mix, with a span of variability in body weight associated with a high prevalence of frailty. Although frailty is usually seen as a wasting disease and significant loss of weight is one of its criteria, frailty can still occur in individuals who are overweight or obese. Therefore, frailty can similarly span across a spectrum through variation in body weights from an anorexic, malnourished (AM) phenotype at one end to a sarcopenic obese (SO) phenotype at the other end. In addition, frailty is associated with a greater loss of the insulin-resistant type II muscle fibres relative to the loss of insulin-sensitive type I fibres, resulting in a reduction in insulin resistance [51]. Hence, each person’s overall insulin resistance will be impacted by variations in body composition across the frailty spectrum. The overall effect of total body weight, muscle mass, the proportion of type II to type I skeletal muscle fibres, and total body fat mass, particularly visceral fat, will determine insulin resistance [52]. Consequently, the SO phenotype is characterised by enhanced insulin resistance because of obesity and an increase in visceral fat, whereas the AM frailty phenotype is characterised by insulin resistance decline because of significant weight loss [53].

## 4. SGLT-2 Inhibitors and GLP-1RA

**Figure 1:**
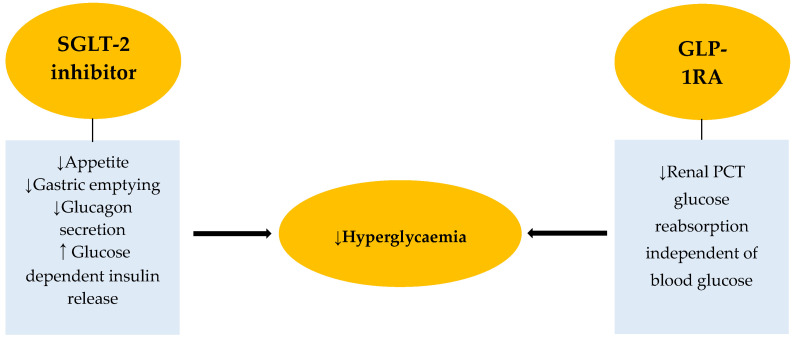
The novel mechanisms of action of SGLT-2 inhibitors and the GLP-1RA lower their risk of inducing hypoglycaemia. SGLT-2—sodium glucose cotransporter, GLP1-RA—glucagon like peptide receptor agonist, PCT—proximal convoluted tubule.

By decreasing glucose reabsorption in the kidneys’ proximal convoluted tubules, SGLT-2 inhibitors lower blood glucose levels. This effect leads to diuresis and natriuresis, which lowers blood pressure. GLP-1RA decreases glucagon secretion, delays stomach emptying, increases glucose-dependent insulin secretion, and centrally suppresses hunger. Because of their novel mechanisms of action, these new therapies have minimal risk of hypoglycaemia (Figure 1).

### 4.1. Current Use

There is uncertainty and less use of SGLT-2 inhibitors and GLP-1RA in care homes. In a comprehensive care home cross-sectional study in the US, which included 141,636 residents with diabetes, only 1018 (1.1%) residents used GLP-1RA and 569 (0.6%) used SGLT-2 inhibitors. Compared to people receiving other agents, a higher percentage of GLP-1RA users were overweight (60.5% vs. 33–42% as monotherapy and 76.2% vs. 50–58% oral therapy in combination with basal insulin). Metformin was the most commonly used agent in 36,383 (35.5%) residents. The study highlighted the alarmingly high use of sulfonylurea (SU) in 20,054 (19.6%) residents, increasing their risk of hypoglycaemia [11]. Another US care home cross-sectional study, which included 71,200 to 120,861 residents ≥ 65 years old with type 2 diabetes mellitus each year from 2016 to 2020, found that metformin, SU, and dipeptidyl peptidase-4 (DPP-4) inhibitors were the most prescribed medications. SGLT-2 inhibitors were prescribed only to ≤4% of patients. The injectable regimen most frequently prescribed was basal plus prandial insulin. GLP-1RA alone was rarely prescribed (0.4–1%), while in 2016 and 2020, 1% and 6% diabetes patients, respectively, received GLP-1RA along with insulin. Prescription trends were largely stable between 2016 and 2020. Oral and injectable glucose-lowering prescription drug usage by year was consistent across age groups, comorbidities (<3 or ≥3), and body mass index (<30 or ≥30). The prescribing of SGLT-2 inhibitors (≤4% of residents/year) and GLP-1RA (the latter in combination with insulin ≤6%/year) remained infrequent during the study [54]. A retrospective US study described the adoption of SGLT-2 inhibitors by prescribers caring for long-stay care home residents, compared with SU. For 117,667 care home residents aged 65 years or older, 36,427 unique prescribers (SGLT-2 inhibitors, 5811; SU, 35,443) were identified. Most of the prescribers (87%) prescribed SU alone, 2% prescribed SGLT-2 inhibitors as monotherapy, and 11% prescribed both. Compared with general physicians, geriatricians were the least likely to prescribe SGLT-2 inhibitors. However, there was some increase in the number of residents using SGLT-2 inhibitors from 2344 in 2017 to 5748 in 2019, a proportional increase from 3% to 10% (*p* = 0.001). The study suggested that the low use of SGLT-2 inhibitors in nursing home residents by geriatricians could be related to the lack of evidence of benefits and fear of side effects in these multimorbid patients with short life expectancy [55]. Advantages and disadvantages of the new therapy are summarised in Table 1.

### 4.2. Efficacy

Although the cardio-renal protection of SGLT-2 inhibitors and GLP-1RA extends to include the elderly ≥ 65 years, there is no direct evidence that their favourable effect extends to include frail elderly with diabetes or people residing in care homes [56]. This is largely because these populations have rarely been recruited for or have been totally excluded from clinical trials. The current little evidence is indirect from retrospective or post hoc analyses of previous clinical trials on frail community patients, but not care home residents. The Dapagliflozin Evaluation to Improve the Lives of Patients with Pre-served Ejection Fraction Heart Failure (DELIVER) research, for instance, demonstrated that highly frail patients gained more from dapagliflozin. For fragile, more frail, and most frail subjects, the hazard ratio (HR) for the major end point (time to a first heart failure exacerbation episode or CV mortality) was 0.85 (95% CI, 0.68 to 1.06), 0.89 (0.74 to 1.08), and 0.74 (0.61 to 0.91), respectively [57]. After a median follow-up of 2.3 years, the number needed to treat (NNT) to avoid one primary event was 40 for non-frail participants, 31 for more frail people, and 19 for the most frail subjects [57]. Furthermore, regardless of frailty level, dapagliflozin decreased CV death or HF worsening, and the benefits were higher with a worse frailty state, as reported by the post hoc analysis of the Dapagliflozin and Prevention of Adverse Outcomes in Heart Failure (DAPA-HF) trial. From the lowest to the greatest frailty class, the difference in the incident rate per 100-person-years for dapagliflozin versus placebo was −3.5 (95% CI −5.7 to −1.2) for non-frail, −3.6 (−6.6 to −0.5) for more frail, and −7.9 (−13.9 to −1.9) for the majority of frail participants during a median follow-up of 18.2 months. From the lowest to greatest frailty classifications, the NNT to prevent one incident per 100 person-years was 31, 25, and 15, respectively [58]. Similarly, in a retrospective analysis, the absolute benefits of such therapy, in comparison to DPP-4 inhibitors, were greater in frail compared with non-frail subjects [59]. An important observation in the above studies is that up to a third of the frail older people with diabetes included were either overweight or obese [57,58,59]. Furthermore, the prevalence of metabolic syndrome and a history of CV events was more common in the frail subjects. Obesity-related conditions such as obstructive sleep apnoea and gastro-oesophageal reflux disease were also more prevalent in frail participants, confirming established obesity-related complications in this cohort. Other studies similarly showed that the beneficial effects of the new therapy in frail elderly individuals with diabetes were demonstrated in participants who were either obese or overweight [60,61] (Table 2).

### 4.3. Safety

The above studies reported that serious side effects or discontinuation of SGLT-2 inhibitors or GLP-1RA were not more prevalent in frail compared with non-frail participants [57,58,59,60,61]. The SGLT-2 inhibitors in Older Diabetic patients (SOLD) study, which included 739 adults, with a mean (SD) age of 75.4 (3.9) years, found that drug withdrawal occurred in 174 (23.5%) patients. Infections of the genitourinary tract were the most frequent reason (44.1% at 6 months and 41.7% at 12 months), followed by general non-tolerance (16.6% at 6 months and 20.8% at 12 months), such as nausea, reduced appetite, and excessive diuresis. At six and twelve months, 0.8% and 12.5%, respectively, experienced acute kidney injury (AKI). In the first six months, 11% experienced symptoms of volume loss, including low blood pressure, postural hypotension, pre-syncope, and syncope. Compared to patients who continued taking their medications, those who stopped therapy tended to be of older age {mean (SD) age 75.8 (4.2) vs. 74.7 (3.8) years} and had a lower mean (SD) BMI (27.9 (3.3) vs. 29.2 (4.7)). The medication withdrawal rate was almost double in patients ≥ 80 years old compared to younger patients (35% vs. 19.1%). Furthermore, a lower baseline BMI was significantly associated with SGLT-2 inhibitor discontinuation, OR 0.92, 95% CI 0.88 to 0.97, *p* < 0.001 [62]. A retrospective US care home study compared the safety of SGLT-2 inhibitors and GLP-1RA in a large sample of 7710 residents aged ≥ 66 years with type 2 diabetes. SGLT-2 initiators increased the risk of diabetic ketoacidosis (DKA, HR 1.95, 95% confidence limits (CLs) 1.27 to 2.99), death (1.18, 1.02 to 1.36), major adverse CV events (1.16, 0.88 to 1.53), HF (1.21, 0.95 to 1.52), AKI (1.13, 1.00 to 1.28), and urinary tract infection (UTI) or genital infection (1.11, 0.99 to 1.26) compared with GLP-1RA. These estimates were imprecise and compatible with no difference between the treatment groups. Rates of fall-related injuries (0.83, 0.61 to 1.12), all fractures (0.87, 0.64 to 1.18), hip fractures (0.83, 0.50 to 1.37), and hypoglycaemia (0.86, 0.64 to 1.15) were lower in SGLT-2 inhibitors versus GLP-1RA initiators, but estimates were imprecise and compatible with no difference as well. Notably, discontinuation during follow-up was similar between initiators of SGLT-2 inhibitors and GLP-1RA (40%), although the median time to medication discontinuation was longer for GLP-1RA initiators (130 vs. 100 days). Residents who received > 60 days of care in a skilled nursing facility or hospital within 180 days before initiating a drug of interest were excluded. Compared with residents initiating GLP-1RA, those initiating SGLT-2 inhibitors were less likely to have a BMI > 30 kg/m^2^ or to be cognitively intact. The study concluded that SGLT-2 inhibitors are not superior but may have inferior effectiveness compared with GLP-1RA for CV outcomes and mortality in care home subjects. In addition, residents treated with SGLT-2 inhibitors should be monitored closely for DKA [63].

### 4.4. Scope of Use

It appears that diabetes prevalence in care homes is increasing, and this population is characterised by high levels of comorbidity and frailty. Therefore, it is at increased risk of hypoglycaemia, visits to the emergency department, hospitalisation, and reduced quality of life. SGLT-2 inhibitors and GLP-1RA therapy are associated with minimal risk of hypoglycaemia, which makes them appealing choices in elderly people residing in care homes. Furthermore, their beneficial effects are independent of HbA1c, and tight glycaemic control is not required to achieve these benefits. As a result, overtreatment to achieve glycaemic targets is not an expected complication nor required. In a US study, overtreatment was common in Veterans Affairs (VA) care home residents. The study included 7422 subjects, with a mean (SD) age of 74.6 (7.9) years, with type 2 diabetes mellitus and found that 40% of residents were either overtreated or potentially overtreated. In addition, 31.7% experienced a minimum of one episode of hypoglycaemia (<3.9 mmol/L) [64]. A systematic review of global care homes found wide variations in the rates of overtreatment and undertreatment of residents with diabetes (5–86% and 1–35%, respectively) [65]. Clinical trial evidence suggests that glycaemic control did not improve micro or macrovascular outcomes in elderly people with diabetes but increased hypoglycaemia risk by 1.5–3-fold [66]. Thus, rather than glycaemic control, the new therapy’s range of application should be determined by its prognostic benefits. Residents in care homes with type 2 diabetes have a raised baseline risk and are most likely to benefit from the new treatment because they have higher rates of morbidities, such as hypertension, cardiovascular illness, and chronic kidney disease. (Box 2) The goals of therapy in care home settings should also focus on reducing extreme blood glucose variability to avoid decompensation, hospitalisation, and poor life quality. In addition, the new therapy has extra glycaemic effects, such as diuretic and hypotensive effects. These extra effects may help reduce the need for other medications and improve polypharmacy.

Box 2 **Reasons to consider use of SGLT-2 inhibitors and GLP-1 RA in care home residents with type 2 diabetes mellitus.**

- High comorbidity burden
- High hypoglycaemic burden
- Increased prevalence of cardiovascular disease
- Increased prevalence of chronic kidney disease
- Increased prevalence of heart failure
- Increased prevalence of hypertension
- Increased prevalence of stroke
- Increased prevalence of peripheral vascular disease
- Increased polypharmacy
- Increased prevalence of obesity
- Increased prevalence of dyslipidaemia
- Increased prevalence of metabolic syndrome
- High atherosclerotic cardiovascular risk

### 4.5. Suitable Patients

**Figure 2:**
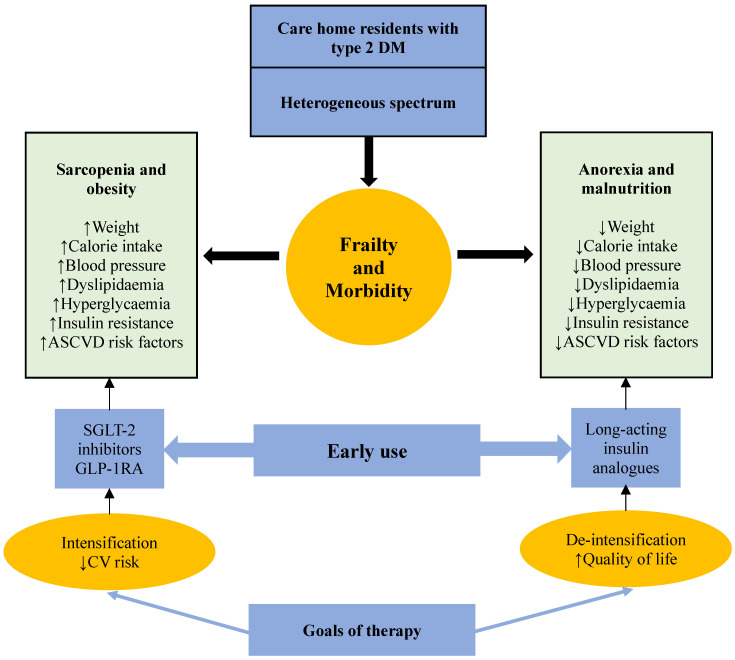
Selection of patients suitable for the use of SGLT-2 inhibitors and GLP-1RAs among care home residents with type 2 diabetes mellitus. Patients living in care homes have a heterogeneous mix of body composition, which impacts their underlying insulin resistance and cardiovascular risk. SGLT-2 inhibitors and GLP-1RAs should be considered early in sarcopenic patients with obesity, while long-acting insulin analogues are more suitable in those who are anorexic and malnourished. DM—diabetes mellitus, ASCVD—atherosclerotic cardiovascular disease, SGLT-2—sodium glucose co-transporter, GLP-1RA—glucagon like receptor agonist, CV—cardiovascular.

The current guidelines on care home diabetes are general and do not actively specify which patients should prognostically benefit from the new therapy. They are passively more concerned and focused on tolerability and avoidance of side effects (Table 3) [5,6,67,68,69]. Frailty, comorbidities, and a diverse body composition are prevalent in elderly individuals with diabetes residing in care homes. Depending on the net effect of body weight, ratio of visceral fat to lean muscle mass and the dominant muscle fibre type, this variation leads to a spectrum of diverse insulin sensitivity. Patients with obesity stand to tolerate and to gain most from the new treatment because they have an unfavourable metabolic profile that includes persistent hyperglycaemia, dyslipidaemia, hypertension and increased insulin resistance. The scant available research, thus far, has primarily demonstrated advantages for elderly individuals with diabetes who are obese or, at least, overweight. Furthermore, these medicines start to have protective benefits in the first few months of treatment. Therefore, these patients will still gain from such therapy even if their life expectancy is short. These new agents, although initially developed as a hypoglycaemic therapy, have anti-metabolic syndrome properties that improve the whole metabolic profile and improve cardiovascular risk beyond glycaemic control. For example, these agents improve hepatic functions and reduce fat accumulation in the liver in patients suffering from non-alcoholic fatty liver disease (NAFLD) or non-alcoholic steatohepatitis (NASH), which has recently been renamed as metabolic dysfunction-associated fatty liver disease (MAFLD) [70,71]. For care home patients with diabetes who are obese, an approach that includes early introduction of the new agents combined with intensification of therapy and multifactorial intervention to reduce cardiovascular risk is suitable. However, this approach should be individualised, considering tolerability. Such therapy is not suitable for frail residents who are anorexic and malnourished with significant weight loss due to increased risk of further loss of weight, dehydration, hypotension, and falls, which may lead to fractures. Compared to people living in the community, residents of care homes experience ten times as many falls and fractures. Consequently, with this frailty phenotype, special focus should be given to risk avoidance [72]. Significant weight loss may lower insulin resistance and return HbA1c to normal in the AM frailty phenotype [47]. Therefore, for care home patients with diabetes who are anorexic and malnourished, an approach that avoids these new agents and aims for therapy deintensification and life quality is reasonable (Figure 1 and Figure 2).

## 5. Personalised Approach

**Figure 3:**
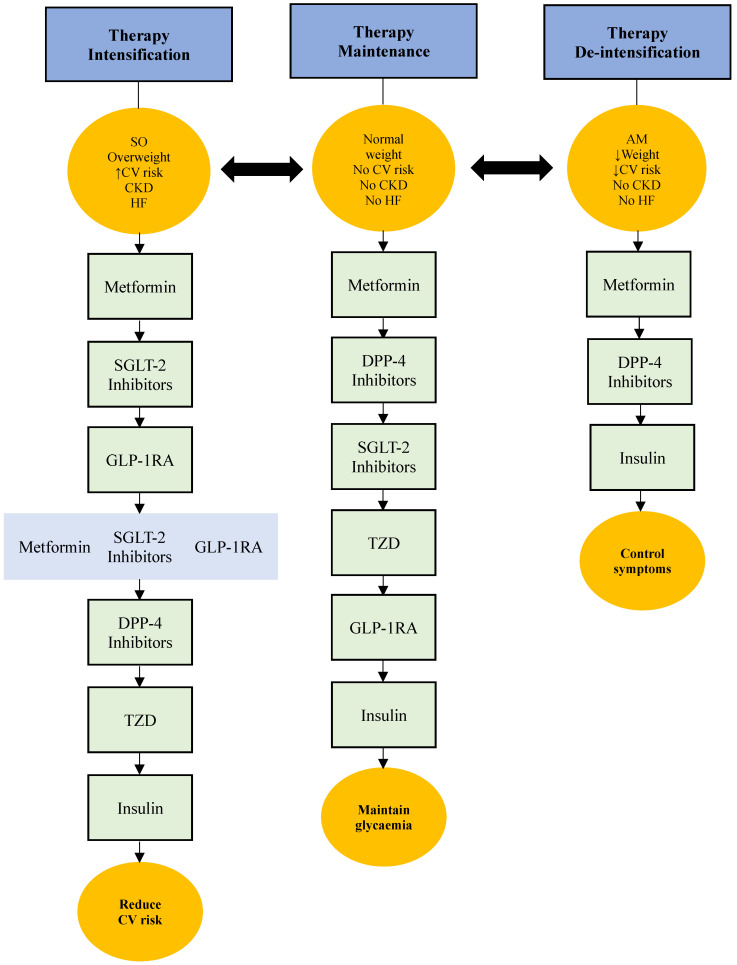
Suggested algorithm for use of hypoglycaemic therapy in care home residents based on their phenotype and cardiovascular risk. Frail SO patients with high CV risk will benefit from early introduction of triple therapy of metformin, SGLT-2 inhibitors, and GLP-1RAs on a case-by-case basis according to tolerability. Normal weight or low-CV-risk patients can start on oral therapy with gradual titration based on glycaemic control. In AM frail patients with weight loss, early introduction of long-acting insulin is a good option for its anabolic properties. Sulfonylureas or glinides are better avoided in care home residents due to high risk of hypoglycaemia. SO—sarcopenic obesity, CV—cardiovascular risk, CKD—chronic kidney disease, HF—heart failure, AM—anorexic and malnourished, SGLT-2—sodium glucose cotransporter, DPP-4—dipeptidyl peptidase, GLP-1RA—glucagon like peptide receptor agonist, TZD—Thiazolidinedione.

It appears that residents with type 2 diabetes in care homes are characterised by high burdens of morbidities, frailty, and polypharmacy and are at risk of hypoglycaemia. They are also heterogeneous with a wide spectrum of body compositions, which leads to a wide variation in insulin sensitivity and their cardiovascular risk. The available little evidence for the use of SGLT-2 inhibitors and GLP-1RAs in frail elderly is indirect and suggests benefits in the obese or overweight end of the frailty spectrum. This evidence is extrapolated from frail elderly people living in the community, as there are no studies that have included sufficient participants from care homes. Because of various obvious reasons, there will unlikely be any clinical trials in this vulnerable population. Current prescriptions for SGLT-2 inhibitors and GLP-1RAs in care homes are suboptimal, and there is a scope for personalising their use if the right patient is selected. Furthermore, the inappropriate use of hypoglycaemic agents with a high potential of causing hypoglycaemia and inappropriately tight glycaemic control are still common in care homes. A personalised approach is to base the prescription of glucose-lowering agents on the underlying atherosclerotic cardiovascular (ASCV) risk, frailty phenotype, and potential tolerability. A triple first-line choice of metformin, SGLT-2 inhibitors, and GLP-1RAs is appropriate in SO frail patients who have high ASCV risk. Although guidelines do not specifically suggest triple therapy in frail subjects, its use in high ASCVD risk patients is recommended [5,6]. The use of this therapy, however, should be tried case by case, depending on tolerability and engagement with exercise, to reduce the risk of sarcopenia. Both groups of therapy have an additive and complementary effect, with SGLT-2 inhibitors being superior in cardio-renal outcomes, while GLP-1RAs improve dyslipidaemia and unfavourable metabolism better [73]. A meta-analysis demonstrated that the combined therapy reduced HbA1c, systolic blood pressure, and body weight without increasing the risk of severe hypoglycaemia compared to either agent alone [74]. The initial triple therapy should be initiated in these high-risk patients, independent of HbA1c. If glycaemic control is still required, other oral agents, such as DPP-4 inhibitors and thiazolidines, can be added, but sulfonylurea should be avoided due to the high hypoglycaemia risk [75]. In AM frail care home residents with erratic eating patterns, de-intensification of oral therapy and early introduction of a long-acting insulin analogue is a reasonable choice because of its weight-gaining properties and lower hypoglycaemia risk [76,77] (Figure 3).

## 6. Conclusions

The newly introduced therapy of SGLT-2 inhibitors and GLP-1RAs appears to be underused in care home settings. This is because of a lack of direct evidence from clinical studies, as care home residents are mostly excluded from these trials, and clinicians fear the side effects of these agents. However, the little available evidence from observational studies confirms the benefits of these agents in frail elderly with diabetes, particularly those who are obese or overweight. The demographics of care home residents are heterogeneous, with an increasing prevalence of obesity and increased baseline cardiovascular risk. Therefore, with the increasing prevalence of obesity in these settings, the scope is increasing for the use of this therapy in care homes. Clinicians should act proactively and follow a personalised approach to select suitable patients who will benefit from this therapy on a case-by-case basis.

## 7. Future Perspectives

At the moment, the evidence or guidance on the use of the new therapy of SGLT-2 inhibitors and GLP-1RA in care home residents with type 2 diabetes mellitus is not clear. It is unlikely that future clinical trials will include residents in care homes, as they are largely excluded from such research due to difficulties in recruitment and follow-up, due to older age, multimorbidities, and frailty. Therefore, a personalised approach is required. The new therapy appears to be ideal for frail elderly individuals with diabetes residing in care homes due to its minimal risk of hypoglycaemia and its cardioprotective effects in this population, who have a high baseline risk of cardiovascular disease. In addition, it may reduce polypharmacy due to its multisystemic benefits. Furthermore, these agents may have a positive effect on Parkinson’s disease and cognitive function, which have the potential to reduce the risk of these degenerative conditions [78]. Another important point is whether a reduction in cardiorenal complications by the new therapy will lead to a reduction in frailty and an improvement in physical function. This is not yet clear, but future observational studies to explore these areas are required. The current use of the new therapy is low, although there is a large scope for future use in care homes. Residents in care homes with type 2 diabetes should not be disadvantaged, due to their location, and denied such important therapy. Guidelines need to be explicit in advising clinicians on actively identifying patients who will benefit most from such therapy, rather than just focusing on avoiding side effects. As clinicians are still reluctant to use this therapy in care homes, investigations into the barriers to its use are needed. Frailty does not appear to be one metabolically uniform category. Therefore, future studies on the metabolic phenotypes of frailty, the variations in insulin resistance, and the variability in response to the new therapy are an important area for future research.

**Key points**
The following key points can be drawn from this study:
  - The prevalence of elderly individuals with type 2 diabetes residing in care homes is increasing.
  - The current use of SGLT-2 inhibitors and GLP-1RAs in care homes is suboptimal.
  - The available evidence confirms that SGLT-2 inhibitors and GLP-1RAs are more effective in frail elderly individuals with diabetes, especially those who are overweight or obese.
  - Care home residents are a heterogeneous mix of people with wide variations in body composition.
  - There is an increasing scope for the use of SGLT-2 inhibitors and GLP-1RAs in care homes, as obesity prevalence is increasing in these settings.

## Figures and Tables

**Table 1:** Advantages and disadvantages of SGLT-2 inhibitors and GLP-1RA in care home residents with type 2 diabetes mellitus.

Advantages	Disadvantages
Significant cardiovascular risk reduction including hospitalisation for heart failure.	SGLT-2 inhibitors risk of genitourinary infections.
Significant reduction in chronic kidney disease progression and the need for renal replacement therapy.	SGLT-2 inhibitors risk dehydration, hypotension, and falls.
Low risk of hypoglycaemia similar to placebo, which reduces hypoglycaemia-related hospital visits.	SGLT-2 inhibitors risk of euglycaemic diabetic ketoacidosis.
Body weight reduction in individuals with obesity.	SGLT-2 inhibitors risk initial decline in renal function.
Blood pressure reduction in people with hypertension.	GLP-1RA gastrointestinal side effects and loss of appetite.
Simple administration regimen.	GLP-1RA low risk of pancreatitis and gallstones.
Improves metabolic profile in patients with metabolic syndrome.	GLP-1RA injectable route of administration.
Improves quality of life by reducing hospitalisation risk.	Limited data on effect on muscle mass and risk of sarcopenia.
Benefits occur early after starting therapy, making it suitable for those with limited life expectancy.	Weight loss in malnourished underweight patients.
With their multiple effects, may reduce polypharmacy.	Risk of misuse of weight loss injection in individuals with obesity.
Modest HbA1c reduction even with renal impairment.	Risk of miscalculating missed weekly doses of GLP-1RA.
Potential neuroprotective effects, especially for cognitive function.	Limited data on the effect on frailty.

**Table 2:** Studies investigating benefits of SGLT-2 inhibitors or GLP-1RA in frail older people.

Study	Findings	Frail Patient Criteria
Butt, J.H., et al., DELIVER study analysis, multicentre, 2022 [57].	A. High rate (%) of primary end point in worse frailty: FI class 1, 6.3 (95% CI 5.7 to 7.1); class 2, 8.3 (7.5 to 9.1); class 3, 13.4 (12.1 to 14.7, *p* < 0.001). B. Dapagliflozin reduced primary end points: FI class 1 to 3 (HR, 95% CI): 0.85 (0.68 to 1.06), 0.89 (0.74 to 1.08), 0.74 (0.61 to 0.91), *p* = 0.40. C. Dapagliflozin improved cardiomyopathy scores in patients with greater compared to lower frailty: 4 months score in FI class 1, 0.3 (−0.9 to 1.4); class 2, 1.5 (0.3 to 2.7); and class 3, 3.4 (1.7 to 5.1, *p* = 0.021).	A. Most frail subjects (75%) had DM, mean (SD) age, 72.7 (8.8) y. B. Compared with non-frail subjects, most frail subjects had a mean (SD): BMI, 32.1 (6.2) vs. 28.1 (5.8), *p* < 0.001; dyslipidaemia, 86.8% vs. 41.2%, *p* < 0.001; HbA1c, 7.1% (1.6) vs. 6.2% (1.2), *p* < 0.001; HF, 35.3% vs. 22.7%, *p* < 0.001; gout, 19.2% vs. 3.8%, *p* < 0.001; CKD, 71.8% vs. 29.6%, *p* < 0.001; prevalent (%) HTN, 97.9% vs. 77.1%, *p* < 0.001.
Butt, J.H., et al., DAPA-HF post hoc analysis, multicentre, 2022 [58].	Dapagliflozin effective regardless of FI class. Events rates (%) dapagliflozin vs. placebo from low to high FI class: −3.5 (95% CI, −5.7 to −1.2), −3.6 (−6.6 to −0.5), and −7.9 (−13.9 to −1.9). Absolute rates were higher in most frail subjects.	A. Most frail subjects (75.7%) had DM; mean (SD) age, 69.8 (9.0) y. B. Compared with non-frail, most frail subjects had median (IQR) HbA1c, 6.7 (6.0–7.7) vs. 5.9 (5.6–6.4); mean (SD) BMI, 30.6 (6.1) vs. 26.9 (5.7); gout, 20.3 vs. 5.5%; dyslipidaemia, 88.7% vs. 42.8%; HTN, 95.7% vs. 58.3; CKD, 70.8% vs. 23.7%; HF, 50.4% vs. 34.9%.
Kutz, A., et al., retrospective study, US, 2023 [59].	In comparison to DPP-4i: A. SGLT-2i efficacy outcomes (HR): 0.72 (95% CI 0.69 to 0.75), IRD −13.35 (−15.06 to −11.64). IRD range was −6.74 (−8.61 to −4.87) in non-frail and −27.24 (−41.64 to −12.84) in frail (*p* < 0.01). B. GLP-1RA efficacy outcomes (HR): 0.74 (0.71 to 0.77), IRD -15.49 (−17.46 to −13.52), IRD in the low −7.02 (−9.23 to −4.81) and −25.88 (−38.30 to −13.46) in the high frailty class (*p* < 0.01).	100% of subjects have DM, age ≥ 65 y: A. SGLT-2i frail vs. non-frail were obese, 49.5% vs. 29.5%; overweight, 12.1% vs. 9.5%; HTN, 98.6% vs. 86.2%; hyperlipidaemia, 89.8% vs. 82.7%; CKD, 33.8% vs. 7.8%; NASH/NAFLD, 8.5% vs. 4.6%; HF, 40.4% vs. 2.5%. B. GLP-1RA frail vs. non-frail were obese 57% vs. 37%; overweight, 9.6 vs. 7.9%; HTN, 98.7% vs. 87%; hyperlipidaemia, 89.5% vs. 82.8%; CKD, 44.7% vs. 13.4%; NASH/NAFLD, 7.9% vs. 4.9%; HF, 42.7% vs. 2.4%.
Mayne, K.J., et al., EMPA-KIDNEY post hoc analysis, multicentre, 2024 [60].	A. Empagliflozin associated with risk reduction: 28% CKD progression or CV death (HR 0.72, 95% CI 0.64 to 0.82), 14% hospitalisation (0.86, 0.78 to 0.95). B. Empagliflozin greatest absolute risk reduction was in likely frail subjects with highest risk of hospitalisation.	10 mg empagliflozin daily or placebo randomised to 6609 subjects with CKD; likely frail subjects with the highest risk for hospitalisation were significantly obese, had a mean (SD) BMI of 32.1 (7.1) v 28.3 (6.3) and had more prevalent DM 78% vs. 16%, *p* < 0.001, compared to low-risk, likely non-frail subjects.
Vart, P., et al., RCT, multicentre, 2024 [61].	A. Dapagliflozin associated with end point risk reduction in all frail classes: HR (95% CI) 0.50 (0.33 to 0.76), 0.62 (0.45 to 0.85), and 0.64 (0.49 to 0.83), p-interaction = 0.67. B. Secondary end points and renal outcomes were similar: (decline ≥ 50% in eGFR, ESRD or mortality from renal cause), CV endpoint (CV mortality, hospitalisation for HF) and death from all-cause.	A. 4303 subjects: 1162 (27%) not or mild frailty (FI ≤ 0.21), 1642 (38.2%) moderate frailty (FI 0.211–0.31), 1499 (34.8%) severe frailty (FI > 0.311). B. Severely frail compared to non-frail subjects were older, mean (SD) age 66.4 (9) v 53.9 (13.5) y, *p* < 0.001, had more DM 90.8% v 31.3, *p* < 0.001 and were more obese, mean (SD) BMI 31.9 (6.4) v 26.8 (5), *p* < 0.001.

SGLT-2i—sodium glucose cotransporter-2 inhibitor, GLP-1RA—glucagon like peptide-1 receptor agonist, FI—Frailty index, CI—confidence interval, HR—hazard ratio, DM—diabetes mellitus, y—years, SD—standard deviation, BMI—body mass index, HTN—hypertension, HF—heart failure, CKD—chronic kidney disease, IQR—interquartile range, DPP-4i—dipeptidyl peptidase-4 inhibitor, IRD—incidence rate difference, NASH/NAFLD—non-alcoholic steatohepatitis/non-alcoholic fatty liver disease, CV—cardiovascular, RCT—randomised controlled trial, eGFR—estimated glomerular filtration rate, ESRD—end-stage renal disease.

**Table 3:** Guidelines’ recommendations of GLP-1RA and SGLT-2 inhibitors in care home residents with type 2 diabetes mellitus.

Guidelines	GLP-1RA	SGLT-2 Inhibitors
ADA [5]	Given the gastrointestinal side effects, GLP-1RAs are not suitable in elderly people who have unintentional weight loss, are undernourished, or suffer from gastrointestinal diseases. GLP-1RA should not be used in subjects with chronic constipation, significant or recurring ileus, or obstructed bowels. Patients should be regularly monitored for significant loss of weight.	SGLT-2 inhibitors cause UTI and genital fungal infections, more often in women, which may lead to medication withdrawal. Because SGLT-2 inhibitors increase urinary volume, patients should be monitored for urinary incontinence symptoms. Euglycaemic DKA is a potential side effect, especially in patients with multimorbidity who reside in LTC settings, with infection being the most common trigger. SGLT-2 inhibitors may cause osteoporotic bone fractures and clinicians should minimise use in patients at high fracture risk.
IPS [67]	Patients should be monitored for anorexia, loss of weight. Avoid in cases of severe kidney disease (eGFR < 30 mL/min) and consider reduction of dose in moderate renal impairment (except for Liraglutide).	Monitor for urinary frequency, incontinence, hypotension, genitourinary infections, and loss of volume. Avoid use if eGFR is <60 mL/min, and consider reduction in dose in renal impairment.
EuGMS/EDWPOP [68]	SGLT-2 inhibitors and GLP-1RAs are considered second-line therapy added to metformin to reduce risk of CV events or renal impairment, especially in people with obesity. Their subscription should be balanced with potential adverse events. SGLT-2 inhibitors are not appropriate when moderate to severe frailty is present, or in care home residents with loss of weight. They are associated with UTI risk, fungal infections, loss of volume, low blood pressure, and DKA. Their effects on glycaemic control are fewer in patients with eGFR < 60 mL/min. GLP-1RAs are not appropriate for patients with chronic kidney disease or care home residents with loss of weight.	
ESE [6]	All institutions should follow safety procedures. There is high prevalence of chronic kidney disease, undernutrition, and sepsis, which leads to high hypoglycaemia risk. Treatment with oral agents or basal insulin in elderly people with type 2 diabetes in LTC facilities results in a similar risk of hypoglycaemia, which suggests that low, daily dose basal insulin is enough to achieve safe glycaemic control in older residents. Limit the dose of SGLT-2 inhibitors in patients at risk of dehydration. GLP-1RA is commonly associated with nausea, which could be a problem in patients with little oral intake, especially patients with kidney disease.	
IDF [69]	Management issues in care homes for elderly include poor nutrition, loss of weight, hypoglycaemia risk, vulnerability to infections, and lower limb ulcers. Therefore, safety, comfort, life quality, preventative and proactive approaches are the focus. Elderly individuals residing in care homes are mostly comorbid, disabled, frail, on multiple medications, and have short survival. The recommendation for care in these settings is based on little evidence. SGLT-2 inhibitors can lead to genitourinary infections, volume loss, orthostatic hypotension, and loss of weight, limiting the scope of their use. GLP-1RAs are associated with gastrointestinal adverse events, which may be a problem and loss of weight can be significant in patients with low weight.	

GLP-1RA—glucagon like receptor agonist, SGLT-2—sodium glucose cotransporter, ADA—American diabetes association, UTI—urinary tract infection, DKA—diabetic ketoacidosis, LTC—long-term care, IPS—International position statement, eGFR—estimated glomerular filtration rate, EuGMS—European geriatric medicine society, EDWPOP—European diabetes working party for older people, CV—cardiovascular, ESE—European society of endocrinology, IDF—International diabetes federation.

## Data Availability

No new data were created or analysed in this study. Data sharing is not applicable to this article.
